# The Ala307Thr polymorphism of the follicle-stimulating hormone receptor (FSHR) gene is associated with the dose of recombinant FSH received during IVF/ICSI treatment

**DOI:** 10.5935/1518-0557.20230009

**Published:** 2023

**Authors:** Felipe Dieamant, Claudia Guilhermino Petersen, Laura Diniz Vagnini, Bruna Petersen, Juliana Ricci, Andreia Nicoletti, Camila Zamara, Antonio Helio Oliani, Joao Batista A Oliveira, José G Franco Jr.

**Affiliations:** 1 Center for Human Reproduction Prof Franco Jr, Ribeirao Preto, SP, Brazil; 2 Paulista Center for Diagnosis, Research and Training, Ribeirao Preto, SP, Brazil; 3 Sao Jose do Rio Preto School of Medicine FAMERP, Sao Jose do Rio, SP, Preto, Brazil

**Keywords:** FSH, FSHR gene, Ala307Thr polymorphism, ovarian response, ovarian stimulation

## Abstract

**Objective:**

Follicle-stimulating hormone (FSH) is essential for folliculogenesis, acting through the follicle-stimulating hormone receptor (FSHR) that is present on the membrane of granulosa cells. Polymorphisms in the FSHR gene may lead to an altered pattern of receptor expression on the cell surface or to changes in affinity for FSH. The aim of this prospective study was to detect any association between the follicle-stimulating hormone receptor (FSHR) gene Ala307Thr polymorphism (rs6165) and ovarian reserve, ovarian response or clinical results in IVF/ICSI treatment.

**Methods:**

This prospective cohort study included 450 women who underwent IVF/ICSI cycles. DNA was extracted from peripheral blood, and the Ala307Thr FSHR polymorphism (rs6165) was genotyped using the TaqMan SNP genotyping assay. Participants were divided into three groups according to their Ala307Thr FSHR genotype: Thr/Thr (n:141), Thr/Ala (n=213) and Ala/Ala (n=96). The results were tested for associations with age, anti-Mullerian hormone (AMH) levels, antral follicle count (AFC), total dose of r-FSH, follicle size, number of retrieved oocytes, and clinical outcome of IVF/ICSI cycles. The statistical analyses were performed using Fisher’s exact test and the Kruskal‒Wallis test.

**Results:**

An association between the genotype of the FSHR (Ala307Thr) polymorphism and the dose of r-FSH was observed. Patients with the Ala/Ala genotype received a higher r-FSH dose than patients with the Ala/Thr (*p*=0.0002) and Thr/Thr (*p*=0.02) genotypes. No other correlation was observed.

**Conclusion:**

The Ala/Ala genotype was associated with the use of higher doses of recombinant FSH (r-FSH), suggesting that homozygosis of this allelic variant (Ala) provides lower sensitivity to r-FSH.

## INTRODUCTION

The prevalence of infertility is on the rise, affecting approximately 15 to 20% of couples of reproductive age. This condition has medical, social and even financial implications for couples. With the advent of assisted reproduction techniques such as in vitro fertilization (IVF)/intracytoplasmic sperm injection (ICSI), several causes of infertility have been successfully managed, and thousands of patients have benefited and are still benefiting from this technological evolution. Currently, approximately 5% of all births in developed countries are due to the IVF procedure (Gearhart & Coutifaris., 2010; Altmäe et al., 2011).

The demand for reproductive treatments has increased significantly in recent years, and there is a desire to develop more effective and less harmful protocols for patients. Despite the advances in reproductive medicine, the pregnancy rate remains close to 35% per treatment performed. In IVF/ICSI cycles, the expected results depend primarily on the effectiveness of the controlled ovarian stimulation (COS), a routine procedure that precedes IVF/ICSI, in which exogenous gonadotropins are used to induce the development of multiple ovarian follicles (Macklon et al., 2006; Altmäe et al., 2011).

Protocols of COS involve the use of several gonadotropins, including recombinant follicle-stimulating hormone (r-FSH), which has a fundamental role in this process. Several studies have shown high variability in clinical outcomes between patients undergoing COS using r-FSH. This unpredictable variability in the ovarian response to gonadotropins, especially FSH, is one of the great challenges of assisted reproduction programs, with ovarian responses that can vary from poor to high, resulting in cancellations and complications such as ovarian hyperstimulation syndrome (OHSS). Therefore, the development of tools that make it possible to predict the ovarian response to stimulation is fundamental for the success and safety of IVF/ICSI treatments (Coccia & Rizzello, 2008; Twigt et al., 2011).

Several parameters have been used in the evaluation of the ovarian reserve and as possible predictors of the ovarian response for patients who aim for reproductive therapy, such as the woman’s age, her serum level of anti-Mullerian hormone (AMH) and her antral follicle count (AFC) by transvaginal ultrasound. In addition to these factors, polymorphisms in several genes have been studied to find genetic markers that can predict ovarian reserve and/or ovarian response, including polymorphisms in the follicle-stimulating hormone receptor (FSHR) gene, located on chromosome 2 (de Castro et al., 2004; Loutradis et al., 2008; Morón & Ruiz, 2010; Lalioti, 2011; Boudjenah et al., 2012; Desai et al., 2013, Alviggi et al., 2018)

FSH acts through FSHR, which is present in the plasma membrane of granulosa cells. For some years, the occurrence of polymorphisms in FSHR has been studied and reported in infertile women. Some mutations, such as Ile160Thr, Ala189Val, and Asn191Ile, are associated with complete inhibition of FSHR activity. However, there are numerous single-nucleotide polymorphisms (SNPs) located in the introns, coding region and promoter region of FSHR that are associated with receptor dysfunction, including the Ala307Thr polymorphism. The Ala307Thr polymorphism of the FSHR gene is located in exon 10, in the area that encodes its extracellular domain in the FSH binding area (Achrekar et al., 2009a; Overbeek & Lambalk, 2009; Karakaya et al., 2014). Considering that partial interference in FSHR function may result in variability in FSH action, a better understanding of FSHR polymorphisms would become a useful tool in predicting ovarian response in treatments involving assisted reproduction techniques. However, studies in different ethnic groups have observed conflicting results regarding a correlation between that polymorphisms and FSHR function (Klinkert et al., 2006; Mohiyiddeen et al., 2012; Polyzos et al., 2021; Conforti et al; 2022).

The observation that the polymorphism at position 307 in FSHR affects sensitivity to FSH is extremely relevant, as this region that mediates hormone action has already been shown to be crucial in in vitro events involving the production of cyclic AMP in response to FSH. Although several studies have been published regarding the effect of FSHR polymorphisms on ovarian response, most of those studies included a small number of patients and heterogeneous treatment protocols. Therefore, the available studies could not adequately estimate the real effect of SNPs on ovarian response (Perez-Mayorga et al., 2000; Behre et al., 2005; Achrekar et al., 2009b; La Marca et al., 2013; Trevisan et al., 2014; Alviggi et al., 2018; König et al., 2019; Song et al., 2019).

Considering that different protocols of ovarian stimulation have been used to induce growth in the number of follicles, thereby increasing the number of viable oocytes, and that the ovarian response to FSH depends on the FSHR genotype, identifying polymorphic variants of this receptor, such as at position 307, can be a useful tool to predict individual responses to COS with the use of gonadotropins and can help in the development of individualized protocols in IVF/ICSI programs.

The aim of the present study is to determine whether there is any association between the Ala307Thr polymorphism of the FSHR gene (rs6165) and ovarian response during COS in IVF/ICSI cycles. In addition, we aimed to determine whether there is an association between the Ala307Thr polymorphism of the FSHR gene, ovarian reserve and clinical outcomes in an IVF/ICSI cycle.

## MATERIAL AND METHODS

### Subjects

A cross-sectional study was conducted between 2020 and 2022, with 450 Brazilian women undergoing their first IVF/ICSI treatment in the Human Reproduction Center (CRH) – Prof. Franco Jr. This center provided the data of the tests performed for IVF/ICSI treatment: genotypic markers that do not present additional risk in the treatment routine, since they are obtained through a peripheral blood sample already collected to perform routine tests such as the serum dosage of anti-Mullerian hormone (AMH). The AMH measurement and SNP genotyping were carried out at the Paulista Centre for Diagnosis, Research, and Training (CPDP). The AFC was performed by the mentioned centers.

Inclusion criteria

The inclusion criteria were age ≤37 years; regular menstrual cycle; normal karyotype; the observation of two ovaries on transvaginal ultrasound; and the absence of hydrosalpinx, previous ovarian surgeries, endometriosis, infection, or endocrinological disorders.

The ovarian stimulation protocols that were used for patients undergoing IVF/ICSI cycles were restricted to two models, GnRH antagonist and GnRH agonist protocols.

DNA was extracted from a peripheral blood sample, and the FSHR Ala307Thr polymorphism (rs6165) was genotyped as described below. The results obtained were tested for correlations with the patient’s age, body mass index (BMI), level of HAM, CFA, total dose of rFSH, size of ovarian follicles, number of oocytes collected, and clinical outcomes of IVF/ICSI cycles (ongoing pregnancy rate).

The subjects were dichotomized based on their genotype of the Ala307Thr polymorphism of the FSHR gene: homozygotes (Ala/Ala and Thr/Thr) and heterozygotes (Ala/Thr).

### Ultrasound evaluation

Patients underwent transvaginal ultrasound during the follicular phase in cycles prior to IVF/ICSI. The ultrasound marker used in this study was the AFC. The total number of antral follicles measuring between 2 and 9 mm in both ovaries was used to evaluate these patients.

### Enzymatic assay

Anti-Mullerian hormone measurements were performed in peripheral blood using the second-generation modified kit from Beckman Coulter Inc. (GenII ELISA kit/Beckman Coulter Inc., ref A73818) following the manufacturer’s instructions.

To minimize the risks of error in this assay, the same operator performed all tests, and standard high- and low-level controls were included to certify the validity of the assay. Prior to this research, the average values of the intra-assay and interassay coefficients of variation for this exam were calculated. The values obtained were 3.3% and 6.5%, respectively. The minimum detectable value of AMH was 0.01 ng/mL.

### Genotyping

Genomic DNA was extracted from peripheral blood samples of all subjects following the guidelines of the manufacturer of the QIAamp DNA blood mini kit (Qiagen) extraction kit. Polymorphisms (SNPs) of genes preselected through next-generation sequencing (NGS) were used for genotyping. SNP rs6165 of the FSHR gene was geno-typed by real-time polymerase chain reaction (PCR) using TaqMan assays (Applied Biosystems). The mix for the real-time PCR was composed of 1 µL of genomic DNA (100 ng/µL), 5 µL of Master Mix Universal TaqMan (Applied Biosystems), 0.5 µL of probe and 3.5 µL of DNase-free water. The protocol for amplification had the following steps: denaturation at 95 °C for 10 min, followed by 40 cycles of 92 °C for 15 s and 60 °C for 1 min. The thermal cycler used was the StepOnePlus Real Time PCR machine, which is part of the CPDP permanent material. PCR products were analyzed using TaqMan Genotyper v1.3 (ABI) software.

### End-points

The primary endpoint was the total dose of gonadotropin (r-FSH) required during the first IVF/ICSI cycle.

### Sample size

To calculate the sample size needed, the dose of r-FSH was used as the primary result. Sample size was calculated by performing a comparison between three means±standard deviations. A sample size of 84 subjects in each group had 80% power to detect an increase/decrease of 50% at a significance level of 0.05.

### Statistical analysis

Categorical variables are expressed as percentages, and quantitative variables are expressed as mean and standard deviation. In cases where distribution with a nonnormal pattern was observed, the median and interquartile range were used. In situations where the variables presented distributions with extreme deviations, data transformation was performed on the variables that were included in the linear or logistic regression models. For the comparison of categorical variables, Fisher’s test was used to detect differences between two groups, and the chi-square test was used to detect differences between several groups. For the comparison of unpaired continuous variables, the parametric Student’s t test was used when comparing two groups and analysis of variance (ANOVA) when comparing three or more groups, applying the Bonferroni posttest for multiple comparisons.

Linear and/or multiple logistic regression was used, depending on the nature of the outcome for comparison between groups. For all tests used, a *p* value <0.05 was considered statistically significant. Contingency tables containing the combinations of all analyzed parameters were constructed to classify the ovarian reserve, thus establishing the specificity, sensitivity and agreement between the methods. Data analysis and construction of graphs presented in the results were performed using the StatsDirect version 2.7.9 program.

### Ethical considerations

Written informed consent was obtained from all patients included in this trial. Authorization by the FAMERP Ethics Committee in Research (CAAE 60245216.0.0000.5415).

## RESULTS

Hardy–Weinberg equilibrium

Genotype and allele distributions in the patients and the controls conformed to the expectations under Hardy–Weinberg equilibrium.

### Demographic and ovarian stimulation cycle characteristics

Basic demographic characteristics, such as age, BMI (body mass index), and cause and duration of infertility, were not significantly different between women with different FSHR Ala307Thr (rs6165) genotypes (Table 1).

**Table 1:** Main characteristics of infertile women, according to their FSHR gene Ala307Thr (rs6165) genotype.

Characteristics	FSHR Ala307Thr Genotype				p
	Total	Thr/Thr	Thr/Ala	Ala/Ala	
n (%)	450 (100%)	141 (31.3%)	213 (47.4%)	96 (21.3%)	
Age (years)	34.6±4.2	35.0±3.8	34.5±4.1	34.5±4.7	0.57
BMI (kg/m^2^)	24.3±4.2	24.3±4.0	24.3±4.0	24.4±4.7	0.81
AMH(ng/ml)	1.8±2.2	1.7±2.3	1.9±2.1	1.6±2.1	0.26
AFC (n)	13.0±8.2	12.6±8.7	13.4±7.4	12.3±9.0	0.11
ORPI*	1.1±2.8	1.1±3.4	1.1±1.8	1.2±3.4	0.16
Infertility (%)					0.91
1a	76.9%(346/450)	78.0%(110/141)	76.5%(163/213)	76.0%(73/96)	
2a	23.1%(104/450)	22.0%(31/141)	23.5%(50/213)	24.0%(23/96)	
Time of infertility (years)	4.2±3.3	4.4±3.6	4.0±3.2	4.3±3.0	0.24
Etiology (%)					0.30
Male	36.4% (164/450)	36.2%(51/141)	39.0%(83/213)	31.2%(30/96)	
Idiopathic	31.4% (141/450)	30.5%(43/141)	29.6%(63/213)	36.5%(35/96)	
Endometriosis	14.9% (67/450)	19.2%(27/141)	13.6%(29/213)	11.5%(11/96)	
Tuboperitoneal	10.3% (46/450)	7.8%(11/141)	11.7%(25/213)	10.4%(10/96)	
Male+endometriosis	3.3% (15/450)	2.8%(4/141)	3.3%(7/213)	4.2%(4/96)	
Male+tuboperitoneal	2.2% (10/450)	2.1%(3/141)	1.0%(2/213)	5.2%(5/96)	
endometriosis+ tuboperitoneal	1.1% (5/450)	0.7%(1/141)	1.9%(4/213)	0	
Male+endometriosis+ tuboperitoneal	0.4% (2/450)	0.7%(1/141)	0	1.0%(1/96)	

* ORPI: [AMH(ng/ml) × AFC (n/2-9mm)]/age (years).

### Ovarian stimulation cycle characteristics

An association between the genotype of the Ala307Thr polymorphism and the dose of r-FSH was observed. Patients with the Ala/Ala genotype received a higher r-FSH dose than patients with the Ala/Thr (*p*=0.0002) or Thr/Thr (*p*=0.02) genotype (Table 2).

**Table 2:** Ovarian stimulation cycle characteristics of the study population according to FSHR gene Ala307Thr (rs6165) genotype.

Cycle Characteristics	FSHR Ala307Thr Genotype				p
	Total	Thr/Thr	Thr/Ala	Ala/Ala	
Stimulation protocol (%)					0.29
GnRH agonist protocol	52.0%(234/450)	57.4%(81/141)	49.8%(106/213)	49.0%(47/96)	
GnRH antagonist protocol	48.0%(216/450)	42.6%(60/141)	50.2%(107/213)	51.0%(49/96)	
**Total dose FSH (IU)(µ±SD)**	**2144±988**	**2085±922^a^**	**1946±955^b^**	**2364±1060^a^,^b^**	**^a^0.02** **^b^0.0002**
Total dose LH (IU)(µ±SD)	1064±363	1052±368	1052±377	1107±323	0.28
Time of stimulation (days) (µ±SD)	10.3±2.4	10.3±2.5	10.1±2.4	10.5±2.2	0.17
Fail ovarian stimulation (%)	2.2%(10/450)	1.4%(2/141)	2.3%(5/213)	3.1%(3/96)	0.59
Follicles (n) (hCG day)					
≥10 mm	11.7±7.1	11.4±6.6	12.0±7.4	11.6±7.4	0.86
≥16 mm	5.8±3.3	5.9±3.0	5.9±3.3	5.6±3.7	0.52
≥18 mm	3.7±2.1	3.8±2.2	3.7±2.0	3.6±2.5	0.64
Retrieved oocytes (n)					
Total	8.1±5.2	8.2±5.4	8.1±5.1	8.2±5.4	0.98
Metaphase II stage	6.0±4.3	6.1±4.3	5.9±4.1	6.1±4.8	0.92
Metaphase I stage	1.4±1.7	1.4±1.6	1.4±1.7	1.2±1.8	0.39
Germinal vesicle stage	0.7±1.2	0.7±1.2	0.7±1.1	0.9±1.2	0.20
Failed oocyte retrieval (%)	1.8% (8/450)	2.8% (4/141)	1.9% (4/213)	0	0.26

^a^,^b^ Values within rows with the same superscript letter were significantly different.

The distribution of the other characteristics of the ovarian stimulation cycle, such as the number of follicles on the hCG day, total number of oocytes retrieved, total number of metaphase II oocytes, and failed oocyte retrieval, did not differ by Ala307Thr (rs6165) genotype.

### Clinical outcomes

Clinical outcomes such as implantation rate, pregnancy rate, miscarriage rate, and cumulative live birth rate were not significantly different according to Ala307Thr (rs6165) genotype (Table 3).

**Table 3:** Clinical outcomes of the study population according to FSHR gene Ala307Thr (rs6165) genotype.

Clinical outcomes	FSHR Ala307Thr Genotype				p
	Total	Thr/Thr	Thr/Ala	Ala/Ala	
Fertilization rate (%)	64.5±27.0%	64.5±26.4	64.6±26.5%	64.4±29.9%	0.96
Fertilization fail (%)	5.3% (24/450)	5.7%(8/141)	3.8%(8/213)	8.3%(8/96)	0.23
Freeze all (%)	1.8% (8/450)	0	2.8% (6/213)	2.1%(2/96)	0.11
Embryo transfers (n) (µ±SD)	1.9±0.6	1.9±0.5	1.9±0.7	2.1±0.8	0.24
implantation rate (fresh cycle) (%)	25.3%(200/789)	23.9%(58/243)	26.1%(97/371)	25.7%(45/175)	0.82
implantation rate (frozen cycle) (%)	20.5%(54/264)	20.2%(19/94)	21.9%(30/137)	15.2%(5/33)	0.73
Cumulative implantation rate (%)	24.1%(254/1053)	22.8%(77/337)	25.0%(127/508)	24.0%(50/208)	0.78
Pregnancy rate, patient (%)					
Fresh cycle	33.8% (152/450)	31.2% (44/141)	35.2%(75/213)	34.4% (33/96)	0.79
Cumulative(fresh cycle + frozen cycle)	44.0% (198/450)	43.3% (61/141)	46.5%(99/213)	39.6% (38/96)	0.56
Pregnancy, transfer (%)					
Fresh cycle	38.2%(152/398)	34.6%(44/127)	39.9%(75/188)	39.8%(33/83)	0.65
Cumulative(fresh cycle + frozen cycle)	36.7%(198/539)	34.5%(61/177)	38.1%(99/260)	37.3%(38/102)	0.77
Miscarriage rate (%)					
Fresh cycle	18.4%(28/152)	13.6%(6/44)	22.7%(17/75)	15.2%(5/33)	0.42
Cumulative(fresh cycle+frozen cycle)	19.7%(39/198)	11.5%(7/61)	25.2%(25/99)	18.4%(7/38)	0.22
Cumulative live births, patient (%)					
Fresh cycle	27.6% (124/450)	27.0% (38/141)	27.2%(58/213)	29.2% (28/96)	0.79
Cumulative(fresh cycle+frozen cycle)	35.3% (159/450)	38.3% (54/141)	34.7%(74/213)	32.3% (31/96)	0.56
Cumulative live births, transfer (%)					
Fresh cycle	31.2%(124/398)	29.9%(38/127)	30.8%(58/188)	33.7%(28/83)	0.65
Cumulative(fresh cycle+frozen cycle)	29.5%(159/539)	30.5%(54/177)	28.5%(74/260)	30.4%(31/102)	0.77

## DISCUSSION

FSH is essential for follicular growth in females and spermatogenesis in males. It acts through its specific β-subunit, as its heterodimeric molecule also has an alpha-subunit that is common to other glycoprotein hormones (LH, hCG, TSH). FSH, when in the ovaries, binds to its cognate receptor FSHR, which belongs to the family of G-protein coupled receptors. The interaction with this receptor allows FSH to exert its activity in the female reproductive tract. Initially, folliculogenesis is promoted through estradiol production by the aromatase enzyme system, with granulosa cell growth and induction of LH receptors. In mid-cycle, the peaking of LH and FSH together induces essential actions leading to the rupture of the follicular wall during the ovulatory process. Finally, in the early follicular and proliferative phases, FSH recruits new antral follicles for the next cycle of folliculogenesis. Considering the fundamental role of FSH in the female reproductive tract, especially in folliculogenesis, pharmaceutical models of FSH are used in assisted reproduction treatments with the objective of multifollicular growth. Ovarian follicular activity and ovarian response to exogenous FSH appear to be influenced by specific gene expression of gonadotropins and their receptors (Yoshimura & Wallach, 1987; Yong et al., 1992; Palermo, 2007; Conforti et al., 2019).

Several activating or inactivating variants of FSHR have already been identified. The most clinically relevant inactivating variants are located in exons 7 and 10. The resulting phenotypes are varied, with the most typical clinical manifestations including elevated serum FSH levels, amenorrhea and infertility. The clinical manifestations of inactivating variants of the FSHR gene, unlike activating variants, occur only when present in homozygous or compound heterozygous forms (Orio et al., 2006; Desai et al., 2013). In 2000, Perez-Mayorga et al. (2000) demonstrated that the FSHR genotype plays a fundamental role in the physiological responsiveness of a given tissue to FSH stimulation.

Single-nucleotide polymorphisms located within or near the gene encoding FSHR have been shown to affect its sensitivity and expression to gonadotropins (Wunsch et al., 2005; Nakayama et al., 2006; Busch et al., 2016). Eight polymorphisms are present in the coding region, and only two of them have been extensively studied and confirmed to be related to clinically relevant phenotypes in assisted reproduction treatments. The polymorphisms present at positions p.Asn^680^Ser (rs6166) and p.Thr^307^Ala are related to the ovarian response to FSH stimulation. These two polymorphisms are present in exon 10 and are the predominant isoforms in several populations; therefore, they are the focus of most studies on this topic (Desai et al., 2013).

As FSH is essential in follicular growth, it is used for controlled ovarian stimulation during IVF/ICSI protocols. However, similar protocols of ovarian stimulation with exogenous FSH result in variable ovarian responses, from poor to too strong responses. Several parameters have been tested as markers to predict the ovarian response, such as age, hormonal biomarker (AMH) and ultrasound (AFC). However, the constant challenge for clinicians is to determine the optimal dose of FSH capable of generating a satisfactory and safe ovarian response in ART cycles (Kligman & Rosenwaks, 2001; Nardo et al., 2009; Desai et al., 2013). As a genetic biomarker, FSHR genotype could be useful to predict ovarian response and help clinicians to define the best protocol and dose of gonadotropin for ovarian stimulation. To the best of our knowledge, there are no data on the correlation between the rs6165 polymorphism and the total dose of gonadotropin required during ovarian stimulation.

There are studies supporting the role of the FSH rs6166 variant as a predictor of ovarian response to FSH stimulation. The Ser/Ser variant at position 680 of the FSHR gene was related to decreased ovarian reserve, a higher total dose of gonadotropin required for ovarian stimulation, and a lower number of oocytes collected in IVF/ICSI cycles (Perez-Mayorga et al., 2000; Sudo et al., 2002; Behre et al., 2005; Jun et al., 2006; Alviggi et al., 2016; Alviggi & Conforti, 2022). On the other hand, studies on the FSHR rs6165 SNP (p.Thr307Ala) and its relationship with the ovarian response to FSH stimulation are scarce (Conforti et al., 2022). According to our results, the FSHR (Thr^307^Ala, rs6165) polymorphism was associated with a statistically significant increase in the total dose of r-FSH required during ovarian stimulation.

Some studies suggest that carriers of the Thr/Thr variant at position 307 of the FSHR gene have greater activation of FSHR, so their duration of controlled ovarian stimulation would be shorter than that needed for carriers of Thr/Ala and Ala/Ala variants (Trevisan et al., 2014; Alviggi et al., 2018). In addition to these data, a meta-analysis and two subsequent studies showed that patients carrying the Ala/Ala variant of the FSHR gene (rs6165, p Thr307Ala) produced a smaller number of mature oocytes than those carrying the Thr/Ala and Thr/Thr variants (Achrekar et al., 2010; Yan et al., 2013; Motawi et al., 2017; Alviggi et al., 2018). In contrast to these data, the current study showed no significant difference in the characteristics of the IVF/ICSI cycle—stimulation time in days, total number of oocytes collected, or number of oocytes in metaphase II—between FSHR genotypes.

In a Chinese study (Yan et al., 2013) including 450 women who were categorized according to ovarian response (poor <5 oocytes retrieved; normal 5–14 oocytes retrieved; high >14 oocytes retrieved), the poor ovarian responders included significantly more Ala/Ala carriers than Thr/Thr or Thr/Ala carriers (*p*<0.001). This corroborates the findings of the present study, in which the Ala/Ala women required higher doses of r-FSH.

Studies related to the FSHR rs6165 polymorphism have provided limited information regarding clinical outcomes, although they have found no significant between the Ala/Ala, Thr/Thr and Thr/Ala genotypes (Conforti et al., 2022). These findings are consistent with the results of the present study, in which there was no association between FSHR genotype and implantation rate, pregnancy rate or live birth rate.

In this prospective study, we observed that the homozygous Ala/Ala genotype at position 307 of the FSHR gene is associated with the need for a higher dose of recombinant FSH and therefore probably leads to a decrease in the sensitivity of FSHR to gonadotropins. These data demonstrate that FSHR genotyping could be a useful tool in pharmacogenetics for the clinician to identify patients who, regardless of traditional ovarian reserve tests (e.g., AMH and AFC), may require a greater dose of FSH during ovarian stimulation in the ART cycle.

The availability of pharmacogenetics is important since anthropometric characteristics and ovarian reserve tests are not capable of predicting the ovarian response to stimulation with exogenous FSH. Some patients, instead of demonstrating satisfactory hormonal and ultrasonographic biomarkers of ovarian reserve or adequate ORPI (ovarian response prediction index), have poor ovarian response with a suboptimal number of oocytes obtained and consequently worse prognosis of their ART cycles (Oliveira et al., 2012; Alviggi et al., 2018; Conforti et al., 2019).

## CONCLUSION

In conclusion, the FSHR gene Ala/Ala genotype at amino acid position 307 was associated with the use of higher doses of r-FSH, suggesting that homozygosis of this allelic variant (Ala) provides lower sensitivity to r-FSH. Therefore, FSHR gene genotyping and the identification of Ala307Thr (rs6165) SNPs can be used as an additional tool in the individualization of ovarian stimulation protocols.
